# Recovery-Phase Viral Pancreatitis (RVP): Proposal of a New Clinical Framework Arising During the Natural Course of Viral Infection

**DOI:** 10.3390/jcm15207794

**Published:** 2026-10-09

**Authors:** Yuzuru Ohshiro

**Affiliations:** Department of Internal Medicine, Omoromachi Medical Center, Tokushukai Medical Group, Naha 900-0011, Japan; ooshiro@aol.com

**Keywords:** acute pancreatitis, viral infection, recovery phase, recovery-phase viral pancreatitis, immune-mediated inflammation, two-hit model, immune reconstitution inflammatory syndrome, SARS-CoV-2

## Abstract

We propose recovery-phase viral pancreatitis (RVP) as a new clinical framework for acute pancreatitis arising during natural recovery from viral infection. Although pancreatic injury during acute viral illness has been widely reported, pancreatitis arising after improvement of systemic viral symptoms remains poorly characterized. We conducted a focused narrative review with descriptive case-level analysis of published reports of virus-associated acute pancreatitis. We identified 19 cases of recovery-phase pancreatitis involving multiple unrelated viruses, including SARS-CoV-2, dengue virus, hepatitis A virus, and Epstein–Barr virus, although most cases (13/19, 68%) were associated with SARS-CoV-2. We propose a hypothetical two-hit model in which possible pancreatic viral involvement or antigen persistence during acute infection (Hit 1) is followed by recovery-associated immune recognition and pancreatic inflammation (Hit 2). Though RVP may share pathophysiological features with classical immune reconstitution inflammatory syndrome (IRIS), we believe it should be distinguished from IRIS because it arises during the natural course of viral infection. RVP has been described after hospital discharge and may therefore represent an underrecognized temporal association, in which its relationship to the preceding viral infection may be overlooked. Recognition of RVP may facilitate appropriate diagnosis of pancreatitis arising during recovery from viral infection and provide a framework for future mechanistic investigations.

## 1. Introduction

Acute pancreatitis is most commonly caused by gallstones, alcohol use, and hypertriglyceridemia; however, acute pancreatitis associated with viral infections has long been recognized. Infectious causes account for only a small minority of acute pancreatitis cases overall, and virus-associated pancreatitis has traditionally been regarded as an uncommon and sporadically reported condition [1,2]. Nevertheless, acute pancreatitis has been reported in association with a broad range of unrelated viruses, including mumps virus, coxsackievirus, cytomegalovirus, Epstein–Barr virus, varicella-zoster virus, hepatitis A and hepatitis B viruses, dengue virus, and, more recently, SARS-CoV-2 [1,2,3,4].

Two broad mechanisms have been proposed to explain how viral infection may lead to pancreatic injury. First, some viruses may directly involve pancreatic tissue. Hepatitis B virus antigens have been demonstrated within pancreatic tissue at autopsy, suggesting pancreatic viral localization or replication [5], while experimental and human tissue studies have shown that SARS-CoV-2 can infect cells of the endocrine and exocrine pancreas [6] (Müller et al., 2021). However, the causal relationship between SARS-CoV-2 infection and acute pancreatitis remains debated. Second, pancreatic injury may occur indirectly through immune-mediated mechanisms, including cytokine release and inflammatory responses accompanying viral illness rather than direct pancreatic infection [7,8,9]. These mechanisms are not mutually exclusive and may predominate at different stages of viral illness.

The timing of pancreatitis onset may therefore provide important clues to its underlying mechanism. Most reported cases of virus-associated acute pancreatitis have occurred during active viral illness, concurrently with systemic, respiratory, or other manifestations of infection, a pattern compatible with direct viral cytotoxicity, a concurrent systemic inflammatory response, or both. However, some reported cases show a temporal separation between the systemic manifestations of the preceding viral infection and the onset of acute pancreatitis [8,10,11,12]. This observation raises the possibility that the timing of pancreatitis may itself carry mechanistic significance.

Most previous reports and reviews of virus-associated pancreatitis have focused primarily on identifying the causative virus or describing pancreatic involvement in relation to individual viral infections [1,2,9,13]. Comparatively little attention has been paid to the temporal relationship between the clinical course of the viral illness and the subsequent onset of pancreatitis. In particular, pancreatitis arising during apparent clinical recovery has not generally been considered as a potentially shared temporal phenotype across different viral infections. We therefore examined whether pancreatitis occurring during clinical recovery represents a recurring pattern across different viral infections. This distinction may also be clinically relevant because patients who have apparently recovered from their initial infection—including those who have already been discharged from hospital—may no longer be considered at risk for virus-associated pancreatic complications.

A conceptually related phenomenon has been well characterized in immune reconstitution inflammatory syndrome (IRIS), in which restoration of immune competence—most commonly following initiation of antiretroviral therapy in patients with HIV infection—triggers an exaggerated inflammatory response against persistent or previously subclinical microbial antigens [14,15,16]. IRIS illustrates how inflammation associated with a preceding infection may become clinically apparent during restoration of an effective immune response. Whether a conceptually similar process might occur during the natural recovery from an acute viral infection, without therapeutic immune reconstitution, has not been systematically explored in the context of pancreatitis.

We previously reported a patient with acute pancreatitis developing during corticosteroid tapering in a hepatitis B virus carrier [17]. In that case, transient viral activation during immunosuppression followed by immune restoration during steroid reduction was considered a possible mechanism of pancreatic inflammation. Although pharmacologically induced immune suppression and restoration differ from the natural course of viral infection, this observation provided a clinical precedent for considering whether changes in antiviral immunity during recovery might contribute to the timing of pancreatic inflammation.

To investigate this possibility, we performed a focused narrative review incorporating a descriptive case-level analysis of published reports of virus-associated acute pancreatitis. Rather than restricting the review to specific viral pathogens or defining recovery solely by a predetermined interval after infection, we examined the clinical course of each reported patient and classified cases according to the temporal relationship between viral illness and pancreatitis onset.

Based on the recovery-phase pattern identified through this approach, we propose recovery-phase viral pancreatitis (RVP) as a new clinical framework—rather than an established distinct clinical entity—to describe acute pancreatitis developing during the natural recovery phase or after apparent clinical recovery from a viral infection. This concept was initially presented in a preliminary preprint [18]. In the present review, we further characterize the clinical and temporal features of RVP and propose a two-hit hypothesis as a potential mechanistic framework for recovery-phase pancreatic inflammation.

## 2. Methods

### 2.1. Study Design and Objectives

This study employed a focused narrative review incorporating a descriptive case-level analysis of published reports of acute pancreatitis potentially associated with viral infection. Given the exploratory and hypothesis-generating nature of this review—aimed at identifying a previously undescribed temporal phenotype rather than at quantifying its incidence, prevalence, or association with viral infection—this study was conducted as a narrative review rather than a formal systematic review and was therefore not registered in PROSPERO or a comparable protocol registry. Although a structured case-identification process was used to enhance transparency and reduce selection bias, this review did not adhere to full systematic review methodology, including dual independent screening, formal risk-of-bias assessment, or a preregistered protocol. To avoid preferential selection of cases exhibiting the proposed temporal phenotype, cases were assessed regardless of the timing of pancreatitis onset relative to viral illness.

The primary objective was to determine whether a subgroup of acute pancreatitis characterized by onset during clinical recovery from viral infection could be identified and whether a similar temporal pattern was observed across unrelated viral pathogens. A secondary objective was to explore a potential pathophysiological framework that could explain this recovery-phase pattern. This review was not designed to estimate the incidence or prevalence of recovery-phase pancreatitis or to establish a causal relationship between viral infection and acute pancreatitis.

### 2.2. Literature Search Strategy

The literature search was performed in PubMed/MEDLINE using the terms “acute pancreatitis viral infection case report” without field tags or filters, allowing PubMed’s automatic term mapping. The last search was performed on 20 July 2026 and yielded 671 records; restricting the Entrez date to 20 July 2026 or earlier reproduces the same 671 records. The full query as translated by PubMed is provided in Supplementary Table S4. Because PubMed automatically explodes MeSH terms, the search also captured reports indexed under specific viral diseases (e.g., COVID-19, hepatitis A, mumps, and dengue) subsumed under “Virus Diseases.” No restrictions were applied regarding viral species, geographic region, or publication language. Google Scholar and Web of Science were used to retrieve full texts and for citation searching, but not as primary sources for case identification. Backward citation searching was performed by screening the reference lists of the 26 recovery-phase reports, and forward citation searching was performed by screening articles citing these reports using Google Scholar (“Cited by”) and Web of Science (“Times Cited”); no additional eligible cases were identified (Figure 1).

Retrieved articles were assessed for the availability of sufficient clinical information to permit case-level evaluation. Articles lacking sufficient information to determine whether acute pancreatitis was present or to assess its potential relationship to the preceding or concurrent viral illness were considered non-assessable and were not included in the case-level analysis.

### 2.3. Case Eligibility and Assessment

Reports were considered assessable when sufficient clinical information was available to determine whether acute pancreatitis was present and to evaluate its potential relationship to the preceding or concurrent viral illness. Within assessable reports, individual cases diagnosed with acute pancreatitis were identified and evaluated at the patient level. The diagnoses of acute pancreatitis and viral infection were accepted as reported by the authors of the original reports. For the 26 recovery-phase cases, the diagnostic evidence reported in the original publications (abdominal pain, serum amylase or lipase levels relative to the upper limit of normal, and imaging findings) was additionally extracted and compared with the revised Atlanta criteria (Supplementary Table S3). Similarly, etiologic attribution of acute pancreatitis was based primarily on the assessment of the original authors. When an established non-viral cause was identified and considered by the original authors to be the more likely etiology, the case was classified accordingly. Isolated elevation of pancreatic enzymes without a clinical diagnosis of acute pancreatitis was not considered acute pancreatitis.

For each case, available information regarding age, sex, viral pathogen, clinical manifestations of the viral illness, timing of pancreatitis onset, clinical status at pancreatitis onset, occurrence during hospitalization or after hospital discharge, systemic corticosteroid exposure before pancreatitis onset, alternative causes of acute pancreatitis, and clinical outcome was extracted.

Case identification, eligibility assessment, and subsequent etiologic and temporal classification were performed by a single reviewer.

### 2.4. Etiologic and Temporal Classification

Etiologic classification was based primarily on the attribution made in the original report, supplemented by the clinical information provided in the report. Cases fulfilling the criteria for acute pancreatitis were first classified as potentially virus-associated or as having an established non-viral etiology. Cases in which gallstones, alcohol use, hypertriglyceridemia, hypercalcemia, structural pancreatic disease, trauma, definite drug-induced pancreatitis, or another established cause were considered more likely than the viral infection were classified as having a non-viral etiology. Cases without a more likely alternative cause were considered potentially virus-associated acute pancreatitis. This designation does not imply that the pancreatitis was virus-induced; it indicates only that the original authors did not consider another cause more likely, including in cases with reported medication exposure, mild hypertriglyceridemia, polypharmacy, or co-infection. When an additional or intervening illness besides the index viral infection was documented in the original report, this information was incorporated into the etiologic assessment; however, systematic ascertainment of unreported interval exposures, including possible viral co-infection around the time of pancreatitis onset, was not possible from published case-report data for all cases. As described in Section 3.2, we additionally performed a targeted re-review of the original source reports, achieving full-text verification for all 26 recovery-phase cases, specifically to check for documented bacterial, fungal, or additional viral co-infection; because antimicrobial agents were often administered as part of routine treatment protocols or empirical supportive care, cases were classified as having probable co-infection only when the original authors documented an actual clinical suspicion of superinfection, rather than whenever antibiotics had been given.

Potentially virus-associated cases were subsequently classified according to the temporal relationship between viral illness and pancreatitis onset. Acute-phase pancreatitis was defined as pancreatitis developing simultaneously with or during worsening of systemic viral symptoms. Recovery-phase pancreatitis was defined as pancreatitis developing after partial or complete clinical improvement of the viral illness, or after a clinically quiescent or post-acute interval following the preceding infection, including cases occurring after resolution of systemic symptoms or after hospital discharge. Cases in which the temporal relationship could not be reliably determined from the available information were classified as temporally indeterminate. No minimum interval between clinical improvement and pancreatitis onset was required. Classification was based on the clinical status at pancreatitis onset as described by the original authors; when the clinical course was fluctuating, or the timing was ambiguous, the original authors’ description was followed without further inference. Time intervals were recorded as stated in, or directly derivable from the clinical sequence reported in, the original reports and were otherwise recorded as not reported (NR).

For recovery-phase cases, systemic corticosteroid exposure before pancreatitis onset was additionally recorded because of its potential influence on host immune responses. Recovery-phase cases without preceding systemic corticosteroid exposure constituted the natural-course RVP group, whereas those with preceding systemic corticosteroid exposure were analyzed separately as steroid-associated recovery-phase pancreatitis.

## 3. Results

### 3.1. Case Identification and Etiologic Classification

The PubMed search identified 671 articles (Figure 1). Of these, 647 contained sufficient clinical information for case-level assessment and were included in the analysis, whereas 24 were considered non-assessable because of insufficient clinical information. Acute pancreatitis was identified in 677 cases from 513 articles. The remaining 134 assessable articles did not contain a case of acute pancreatitis.

Among the 677 cases, 331 were classified as potentially virus-associated acute pancreatitis, whereas 346 were attributed to established non-viral etiologies.

Among the 331 potentially virus-associated cases, 258 developed pancreatitis during the acute phase of viral illness, 26 developed pancreatitis during the recovery phase, and 47 could not be reliably classified as acute-phase or recovery-phase because of insufficient information regarding the temporal relationship between viral illness and pancreatitis onset.

Among the 26 recovery-phase cases, 7 had received systemic corticosteroids during the preceding acute viral illness. Because corticosteroid exposure may alter antiviral immunity and complicate interpretation of the natural recovery process, these cases were analyzed separately as steroid-associated recovery-phase pancreatitis. The remaining 19 cases, in which recovery-phase pancreatitis occurred without preceding systemic corticosteroid treatment, constituted the natural-course RVP group (Table 1 and Table 2).

### 3.2. Natural-Course Recovery-Phase Viral Pancreatitis

Natural-course RVP was identified in 19 cases without preceding systemic corticosteroid exposure (Table 1). These cases involved multiple unrelated viral infections, including SARS-CoV-2, hepatitis A virus, hepatitis E virus, Epstein–Barr virus, dengue virus, and mumps virus.

Despite differences in the preceding viral illnesses, pancreatitis developed after the acute viral phase had subsided or become clinically quiescent. The clinical course included patients with partial improvement, complete resolution of preceding symptoms, convalescent viral illness, and clinically quiescent infection. Several patients developed pancreatitis while recovering at home, and some developed pancreatitis after hospital discharge and required readmission.

The interval between viral illness onset and pancreatitis was generally within several weeks, although the precise interval could not be determined in all cases. The detailed temporal relationship between the preceding viral illness and pancreatitis onset is summarized in Table 1.

Of the 19 natural-course RVP cases, 13 (68%) were associated with SARS-CoV-2, and the remaining 6 cases were distributed across five other viruses (hepatitis A virus, *n* = 2; hepatitis E virus, *n* = 1; Epstein–Barr virus, *n* = 1; dengue virus, *n* = 1; mumps virus, *n* = 1). In a sensitivity analysis restricted to the 6 non-SARS-CoV-2 cases, the same recovery-phase pattern was observed, with pancreatitis onset occurring approximately 2 weeks to 1 month after the onset of the preceding viral illness, suggesting that the recovery-phase temporal pattern was also observable outside SARS-CoV-2-associated cases. Because of the small number of non-SARS-CoV-2 cases, however, this finding should be interpreted cautiously, and confirmation across a broader range of viral pathogens will require additional cases.

To further address the possibility of confounding co-infection, we conducted a targeted re-review of the original source reports for evidence of a documented bacterial, fungal, or additional viral infection around the time of the antecedent illness, achieving full-text verification for all 26 recovery-phase cases (19 natural-course and 7 steroid-associated). Because antimicrobial agents were frequently given as part of routine COVID-19 treatment protocols or empirical supportive care, we distinguished cases in which the original authors documented an actual clinical suspicion of bacterial or fungal superinfection from cases in which antibiotics were administered empirically without such documented suspicion; only the former were classified as probable co-infection.

A documented or probable bacterial or fungal co-infection was identified in 5 of the 26 cases (19%). Confirmed, culture- or sequencing-based co-infection was documented in four cases: *Streptococcus pneumoniae* pneumonia (Anand et al. [10]); a *Staphylococcus aureus* respiratory superinfection confirmed by sputum culture (Brikman et al. [35]), although the original authors attributed the pancreatitis to SARS-CoV-2 infection, medication used to manage the infection, or a combination of both, rather than to the staphylococcal infection specifically; *Escherichia coli* and *Enterococcus faecium* detected by metagenomic next-generation sequencing of sputum in a patient with chronic pulmonary aspergillosis, a presentation the original authors described as multifactorial, reflecting SARS-CoV-2 infection, potential drug toxicity, altered postoperative anatomy, and severe comorbidity (Yu et al. [39]); and a *Klebsiella pneumoniae* urinary tract infection diagnosed during the same admission as the pancreatitis diagnosis (Ghosh et al. [36]). A further case was classified as possible/probable co-infection because the original authors documented an actual clinical suspicion of bacterial superinfection, with antibiotic therapy escalated in response to progressively increasing inflammatory markers in a patient with chest crepitations (Schembri Higgans et al. [25], Case 2). By contrast, in four cases antibiotics were given as part of routine COVID-19 treatment protocols or empirical management of a new presentation, without the original authors documenting a specific clinical suspicion of bacterial or fungal superinfection; these cases (Sharma et al. [31]; Almutairi et al. [12]; AlHarmi et al. [34]; Razafindrazoto et al. [29]) were therefore classified as showing no documented co-infection, since empirical antimicrobial use alone was not treated as evidence of infection.

The original authors additionally proposed a possible non-infectious alternative contributor in several cases—mild hypertriglyceridemia with long-term statin use (Schembri Higgans et al. [25], Case 2), polypharmacy involving multiple drugs potentially associated with pancreatitis (AlHarmi et al. [34]), drug-induced pancreatitis attributed to dexamethasone and remdesivir (Yamamoto et al. [38]), and, in the case with confirmed staphylococcal co-infection above, a combination of at least four pancreatitis-associated drugs plus tocilizumab (Brikman et al. [35])—none of which met the definite-alternative-etiology threshold used as an exclusion criterion for Table 2.

One case (Agarwal et al. [19]) showed a weakly reactive anti-hepatitis E virus IgM that the original authors considered of no clinical significance; because a concomitant viral exposure could not be excluded with certainty, this case was treated conservatively in the sensitivity assessment below. Individual-level co-infection data were not reported in the original source for two cases (Pinte & Baicus [28]; Suchman et al. [32], the latter a registry-based cohort report without patient-level infectious workup). The remaining 18 of 26 cases (69%), including Sharma et al. [31], Almutairi et al. [12], and AlHarmi et al. [34] as reclassified above, showed no documented bacterial, fungal, or additional viral co-infection based on the clinical, microbiological, and serological information reported in the original source (Amé et al. [20], Case 2; Cheung et al. [21]; Deniel et al. [22]; Garty et al. [23]; Hammami et al. [24]; Jeelani et al. [11]; Meireles et al. [26]; Nguyen & Brophy-Williams [27]; Rajesh et al. [1], Cases 3 and 5; Razafindrazoto et al. [29]; Samies et al. [30], Case 3; Salehi et al. [37]; Wifi et al. [33]; Yamamoto et al. [38]). No definite additional viral co-infection was identified in any case, although a concomitant HEV exposure could not be excluded with certainty in the Agarwal et al. [19] case, as noted above.

As a sensitivity analysis addressing this possibility, restricting the natural-course and steroid-associated cohorts to the 18 cases with no documented or probable co-infection based on the patient-level information available in the original reports showed that the recovery-phase temporal pattern remained observable; this finding does not confirm the proposed phenotype. Because this assessment depends entirely on the completeness of documentation in the original published reports, an entirely unreported interval exposure cannot be excluded with certainty; this residual uncertainty is acknowledged in the Limitations (Section 4.6).

### 3.3. Steroid-Associated Recovery-Phase Pancreatitis

An additional 7 cases developed acute pancreatitis during the recovery phase after systemic corticosteroid treatment during the preceding viral illness (Table 2). These cases were analyzed separately because corticosteroid-induced alterations in antiviral immunity could confound interpretation of the natural recovery process.

As in the natural-course RVP group, pancreatitis developed after documented improvement or resolution of the preceding viral illness, including cases occurring after hospital discharge. However, because of preceding systemic corticosteroid exposure, these cases were not included in the natural-course RVP group.

### 3.4. Temporal Pattern of Recovery-Phase Pancreatitis

Among the 331 potentially virus-associated cases, 258 developed pancreatitis during the acute phase, 26 during the recovery phase, and 47 had an indeterminate temporal relationship. Of the 26 recovery-phase cases, 19 occurred without preceding systemic corticosteroid exposure, and 7 occurred after corticosteroid treatment.

The identification of 19 natural-course RVP cases—predominantly associated with SARS-CoV-2 (13/19, 68%) but also observed, in smaller numbers, across five other unrelated viruses—suggests a recurring temporal phenotype characterized by the emergence of acute pancreatitis after improvement or resolution of the preceding viral illness, although confirmation across a broader range of viral pathogens will require additional cases. The additional steroid-associated cases demonstrated a broadly similar temporal pattern but were analyzed separately because corticosteroid exposure may influence host immune responses.

All 19 natural-course RVP cases fulfilled at least two of the three revised Atlanta criteria; for the steroid-associated cases, the diagnosis of acute pancreatitis was accepted as reported by the original authors (Supplementary Table S3). The interval from viral illness onset to AP could be determined in 21 of the 26 recovery-phase cases and ranged from 6 days to approximately 1 month, mostly 1–3 weeks. The interval from documented clinical improvement or hospital discharge to AP was explicitly reported in only 5 cases (0–7 days) and was not reported in the remaining cases.

## 4. Discussion

### 4.1. Recovery-Phase Viral Pancreatitis as a Proposed Temporal Phenotype

The principal observation of this review is the identification of a subset of patients who developed acute pancreatitis not during worsening of viral illness, but after systemic symptoms had improved or apparently resolved. Among 331 potentially virus-associated cases, 258 developed pancreatitis during the acute phase, 26 during the recovery phase, and 47 had an indeterminate temporal relationship. Of the 26 recovery-phase cases, 19 occurred without preceding systemic corticosteroid treatment; although most of these cases (13/19, 68%) were associated with SARS-CoV-2, the remaining cases spanned five other unrelated viruses. We propose recovery-phase viral pancreatitis (RVP) as a new clinical framework—rather than an established distinct clinical entity—to describe this natural-course temporal phenotype, reflecting a temporal association between viral infection and pancreatitis rather than evidence of direct causality. Accordingly, the designation “potentially virus-associated” reflects the absence of a more likely alternative cause in the original reports and does not imply that the pancreatitis was virus-induced.

The distinction between acute-phase and recovery-phase pancreatitis may be clinically and mechanistically important. Viral pancreatitis has traditionally been discussed primarily in terms of direct viral injury or systemic inflammation during active infection [2,9]. Consistent with these mechanisms, most temporally classifiable cases in our analysis developed pancreatitis during the acute phase of viral illness. However, these mechanisms alone may not fully explain pancreatitis that first becomes apparent after systemic manifestations have subsided. This temporal dissociation between the initial viral illness and subsequent pancreatic inflammation provides the central basis for the concept of RVP.

The predominance of SARS-CoV-2 among natural-course RVP cases (13/19, 68%) warrants comment and likely reflects, at least in part, the disproportionate volume of pancreatitis case reports published during the COVID-19 pandemic rather than a SARS-CoV-2-specific mechanism. Intensive clinical monitoring of patients with COVID-19 and heightened interest in publishing unusual manifestations of COVID-19 may also have increased the recognition and reporting of delayed pancreatitis relative to other viral infections. The same recovery-phase timing was nonetheless also observed, albeit in smaller numbers, in cases associated with hepatitis A virus, hepatitis E virus, Epstein–Barr virus, dengue virus, and mumps virus (Section 3.2), suggesting that the phenotype is not restricted to a single pathogen; confirmation that RVP represents a shared pattern across viral pathogens, rather than a largely SARS-CoV-2-specific observation, will require additional cases. We emphasize that these findings reflect a temporal association between viral infection and subsequent pancreatitis rather than evidence of a direct causal or pathological relationship; the case-report-based design of this review does not permit causal inference.

### 4.2. Proposed Two-Hit Model

Three observations provide the basis for our proposed two-hit model. First, direct pancreatic involvement has been demonstrated or suggested for some viruses, providing biological plausibility for pancreatic viral involvement [5,6]. Second, acute pancreatitis can occur concurrently with systemic viral illness, as supported by the large acute-phase group in our analysis, indicating that viral infection can be associated with pancreatic injury. Third, in natural-course RVP, pancreatitis emerged only after systemic viral manifestations had improved. Together, these observations suggest a temporal separation between possible initial pancreatic viral involvement and subsequent inflammatory injury.

We hypothesize that, during acute infection, viral involvement of the pancreas or persistence of viral components may occur without necessarily producing clinically apparent pancreatitis (Hit 1). As the systemic infection resolves and antiviral immunity develops or recovers, residual pancreatic viral antigens may become targets of immune recognition, resulting in localized inflammation and clinically apparent acute pancreatitis (Hit 2). In this model, the timing of pancreatitis would depend not simply on viral replication itself but on the changing relationship between viral antigen persistence and the host antiviral immune response (Figure 2).

A reported case of measles provides an illustrative clinical observation compatible with this sequence. Takebayashi et al. described a patient with measles encephalitis in whom serum amylase was already mildly elevated before corticosteroid treatment, followed by marked elevations of pancreatic enzymes and clinically apparent pancreatitis as the systemic illness improved [40]. Moreover, measles virus has been demonstrated in inflamed pancreatic tissue in a previous pathological study [41], providing evidence that direct pancreatic viral involvement can occur. Interpretation of the Takebayashi case is complicated by corticosteroid exposure, which the original authors acknowledged as a possible contributor to pancreatitis [40].

The recovery-associated immune recognition proposed in Hit 2 should be distinguished from general post-infectious immune-mediated injury, such as pancreatic autoimmunity. Potential mechanisms underlying delayed, recovery-phase pancreatic inflammation could include molecular mimicry between viral and pancreatic antigens, bystander activation of autoreactive lymphocytes during the resolving inflammatory response, release and presentation of previously sequestered pancreatic antigens following initial tissue injury, or epitope spreading following the initial antiviral immune response. However, because pancreas-specific autoantibodies or autoreactive immune cells were not demonstrated in any of the included cases, autoimmune mechanisms should be regarded as a biologically plausible hypothesis rather than an established mechanism of RVP.

This model remains hypothetical. Neither persistence of pancreatic viral antigens nor recovery-phase activation of virus-specific immune responses has been directly demonstrated in patients with RVP. Moreover, direct pancreatic viral involvement cannot be assumed in individual reported cases. Nevertheless, the model provides a biologically plausible framework for explaining the similar temporal pattern observed across unrelated viral infections and generates testable hypotheses for future studies.

### 4.3. Relationship to Immune Reconstitution Inflammatory Syndrome

RVP is conceptually analogous to—but does not share a demonstrated mechanism with—immune reconstitution inflammatory syndrome (IRIS), a syndrome in which restoration of immune function triggers inflammation directed against persistent or previously subclinical microbial antigens [14,15,16]. Both conditions may therefore involve a temporal dissociation between antigen persistence and the subsequent development or recovery of an effective host immune response. IRIS is invoked here as a conceptual analogy illustrating this general temporal principle, not as evidence for the specific immunological mechanism underlying RVP, which remains unconfirmed. The analogy is therefore limited to the temporal sequence and the proposed mechanism; natural recovery from an acute viral infection in an immunocompetent host differs substantially from immune restoration in a profoundly immunocompromised host.

However, RVP differs fundamentally from classical IRIS. Classical IRIS typically occurs in immunocompromised hosts following therapeutic restoration of immune function [14,15,16], whereas natural-course RVP develops during spontaneous recovery from viral infection without recognized baseline immunodeficiency or deliberate immune manipulation. The clinical settings, implicated pathogens, and circumstances of immune recovery therefore differ (Table 3). RVP may thus share an IRIS-like conceptual framework without representing classical IRIS itself and may instead constitute a distinct recovery-associated phenomenon arising during the natural course of viral infection.

### 4.4. Steroid-Associated Recovery-Phase Pancreatitis

Seven additional cases developed recovery-phase pancreatitis after systemic corticosteroid treatment during the acute viral illness. These cases were analyzed separately because corticosteroid-induced immunosuppression followed by immune recovery may resemble an iatrogenic IRIS-like process rather than natural-course RVP. Nevertheless, their temporal pattern was similar to that observed in the natural-course group.

Our previous report of acute pancreatitis developing during corticosteroid tapering in a hepatitis B virus carrier provides a related clinical example [17]. In that case, viral activation during immunosuppression followed by immune restoration during steroid reduction was considered a possible mechanism of pancreatic inflammation. Although steroid-associated cases do not establish the mechanism of natural-course RVP, they provide a conceptual parallel supporting the possibility that changes in antiviral immunity may contribute to recovery-phase pancreatic inflammation.

### 4.5. RVP as a Potentially Underrecognized Temporal Association

RVP may represent an underrecognized temporal association because pancreatitis develops after improvement of the preceding viral illness, when its relationship to the infection may no longer be readily recognized. In patients who received antiviral agents, corticosteroids, or other medications during the initial illness, subsequent pancreatitis may instead be attributed to drug-induced pancreatic injury. Conversely, when pancreatitis develops after hospital discharge and no conventional etiology is identified, the preceding viral infection may be overlooked and the pancreatitis classified as idiopathic.

Observational studies of COVID-19-associated acute pancreatitis indirectly support the possibility that viral involvement may be underrecognized. Ebik et al. reported that four of seven patients (57.1%) who developed acute pancreatitis during COVID-19 were classified as having idiopathic pancreatitis, compared with 16.8% among patients hospitalized with acute pancreatitis before the pandemic [42]. Other cohorts have also reported relatively high proportions of pancreatitis without an identified conventional etiology among patients with SARS-CoV-2 infection [43,44]. These studies did not evaluate pancreatitis specifically in relation to clinical recovery and therefore cannot establish RVP. Nevertheless, they raise the possibility that a preceding or concurrent viral infection may remain unrecognized as a contributing factor in some patients classified as having idiopathic pancreatitis.

The issue may be particularly relevant after hospital discharge. Several RVP cases in our analysis developed pancreatitis after patients had returned home following improvement of the initial viral illness. At that point, the preceding infection may be considered resolved and unrelated to newly developing abdominal symptoms, particularly if the patient presents to a different physician or institution. Recognition of RVP may therefore help reconnect two clinical events that might otherwise appear unrelated and may reduce misclassification as idiopathic or, in selected cases, drug-induced pancreatitis. However, whether recognition of RVP improves diagnostic accuracy or clinical outcomes remains unknown.

### 4.6. Limitations and Future Directions

Several limitations should be acknowledged. First, this review is based predominantly on published case reports rather than population-based or cohort data, and is therefore subject to substantial selection bias: cases with a clinically striking or unusual temporal pattern, such as pancreatitis emerging after apparent recovery, may be preferentially recognized, investigated, and published, which could inflate the apparent prevalence and consistency of the recovery-phase phenotype. This case-report-based design, together with publication bias, incomplete clinical information, and heterogeneity in diagnostic and etiological evaluation, limits our ability to determine whether RVP reflects a true biological pattern or an artifact of selective reporting. In several cases, alternative etiologies could not be completely excluded despite a temporal pattern compatible with RVP; for example, the original authors raised the possibility of drug-induced pancreatitis in two steroid-associated cases (Section 3.2). Second, most natural-course RVP cases (13/19, 68%) were associated with SARS-CoV-2, and the small number of cases associated with other viruses limits our ability to determine whether the recovery-phase pattern generalizes equally across viral pathogens; this predominance should be considered when interpreting the proposed framework. Third, case reports may not consistently document interval health events between the index viral illness and the onset of pancreatitis. We addressed this directly through a targeted re-review achieving full-text verification for all 26 recovery-phase cases, which identified a documented or probable bacterial or fungal co-infection in 5 cases and a non-infectious alternative contributor (e.g., polypharmacy or drug-induced pancreatitis) noted by the original authors in several further cases (Section 3.2); restricting the cohort to the 18 cases with no documented or probable co-infection based on the patient-level information available in the original reports showed that the recovery-phase temporal pattern remained observable. Nonetheless, because this assessment depends entirely on the completeness of documentation in the original published reports, an entirely unreported interval exposure cannot be excluded with certainty. Fourth, case identification and classification were performed by a single reviewer, which may have introduced subjective classification bias, particularly in determining clinical recovery and excluding alternative etiologies. In addition, case identification relied on a single database (PubMed/MEDLINE), and relevant reports indexed only in other databases may have been missed, although citation searching of the recovery-phase reports identified no additional cases. The interval from clinical recovery to pancreatitis, which is central to the proposed framework, could be determined in only 5 of 26 cases because the date of symptom resolution was rarely documented, and the diagnosis of acute pancreatitis was accepted as reported in the original publications for the remaining potentially virus-associated cases. Fifth, this review demonstrates only a temporal association, not a direct causal or pathological relationship, between viral infection and pancreatitis, and the available literature does not permit estimation of the incidence or prevalence of RVP. Sixth, the proposed two-hit mechanism, including the possible contribution of autoimmune processes such as molecular mimicry or epitope spreading, is inferential; direct evidence of persistent pancreatic viral antigens and recovery-associated pancreatic immune activation is currently lacking. Finally, because the proposed phenotype was identified retrospectively from published reports, the apparent clustering of pancreatitis during recovery may partly reflect reporting or selection bias rather than a distinct biological process.

Future studies should prospectively record the exact dates of viral symptom onset, symptom resolution, hospital discharge, medication initiation and withdrawal, onset of pancreatic symptoms, pancreatic enzyme elevation, imaging abnormalities, and any recurrent episodes, which would allow future investigators to determine whether RVP represents a reproducible phenotype. Serial assessment of viral burden together with virus-specific cellular and humoral immune responses may help determine whether changes in antiviral immunity precede pancreatic inflammation. Demonstration of persistent viral antigen or nucleic acid within pancreatic tissue, together with characterization of local immune responses, would provide particularly important evidence for or against the proposed two-hit model. Independent or consensus-based assessment of temporal classification in future studies would also help establish the reproducibility of the RVP phenotype. Prospective studies would further be required to determine the frequency of RVP and whether recognition of this temporal phenotype improves etiological classification or clinical management.

In conclusion, our analysis identifies a recovery-phase temporal phenotype of acute pancreatitis that was predominantly, though not exclusively, associated with SARS-CoV-2 infection, with smaller numbers of cases observed across several other unrelated viruses. We propose RVP as a new clinical framework—rather than an established clinical entity—and a two-hit model as a potential, hypothesis-generating mechanistic framework linking possible initial pancreatic viral involvement to subsequent recovery-associated immune inflammation; these observations reflect a temporal association and do not establish direct causality. Recognition of this phenotype may be particularly important after hospital discharge, when its relationship to the preceding viral infection can easily be overlooked. When conventional causes of pancreatitis have not been identified, a preceding viral infection should be considered a possible contributor rather than assumed to be causative.

## Figures and Tables

**Figure 1:**
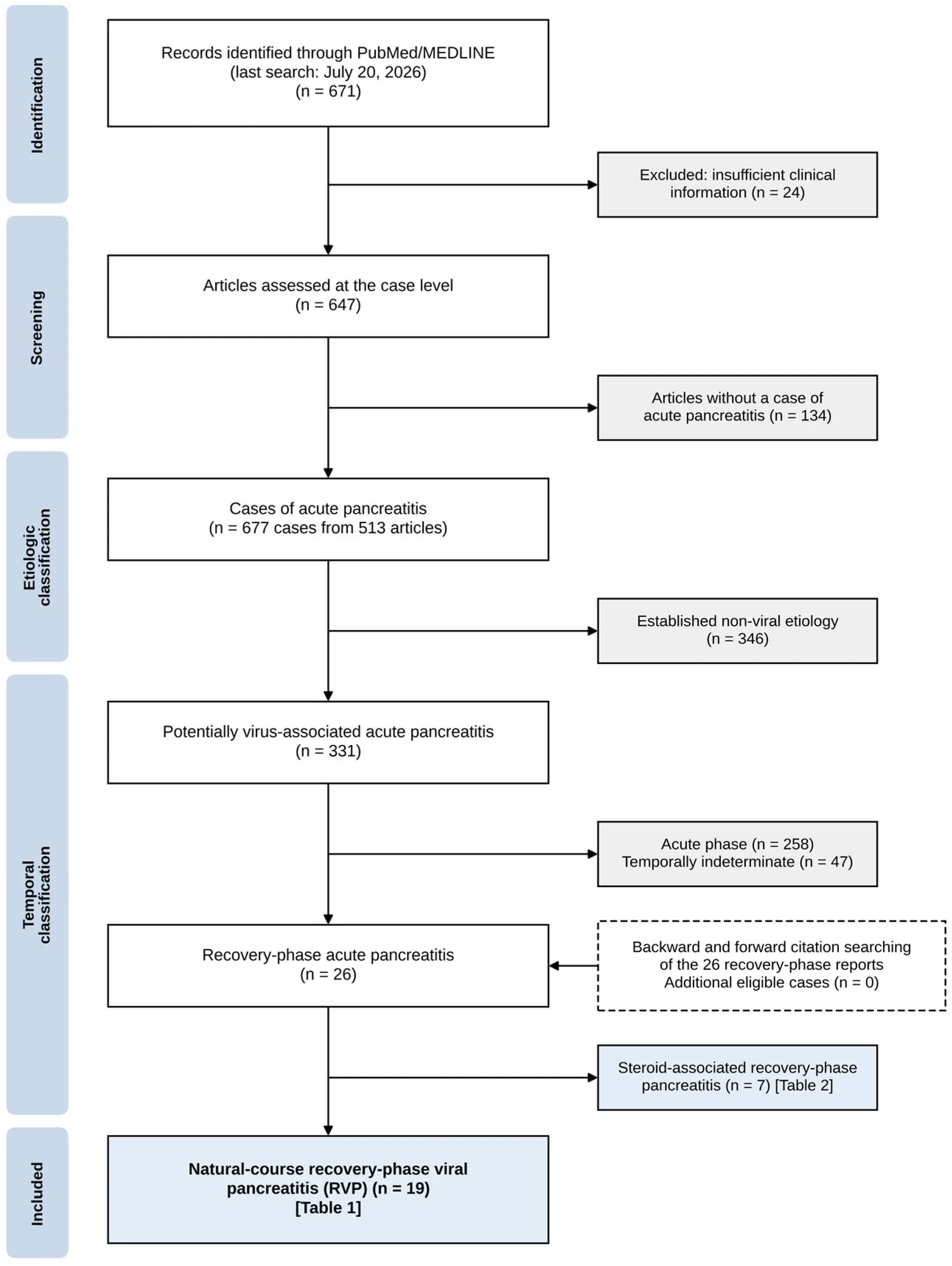
Flow diagram of case identification and classification. Records were identified through a PubMed/MEDLINE search (last search: 20 July 2026). Backward and forward citation searching of the 26 recovery-phase reports identified no additional eligible cases. AP, acute pancreatitis; RVP, recovery-phase viral pancreatitis.

**Figure 2:**
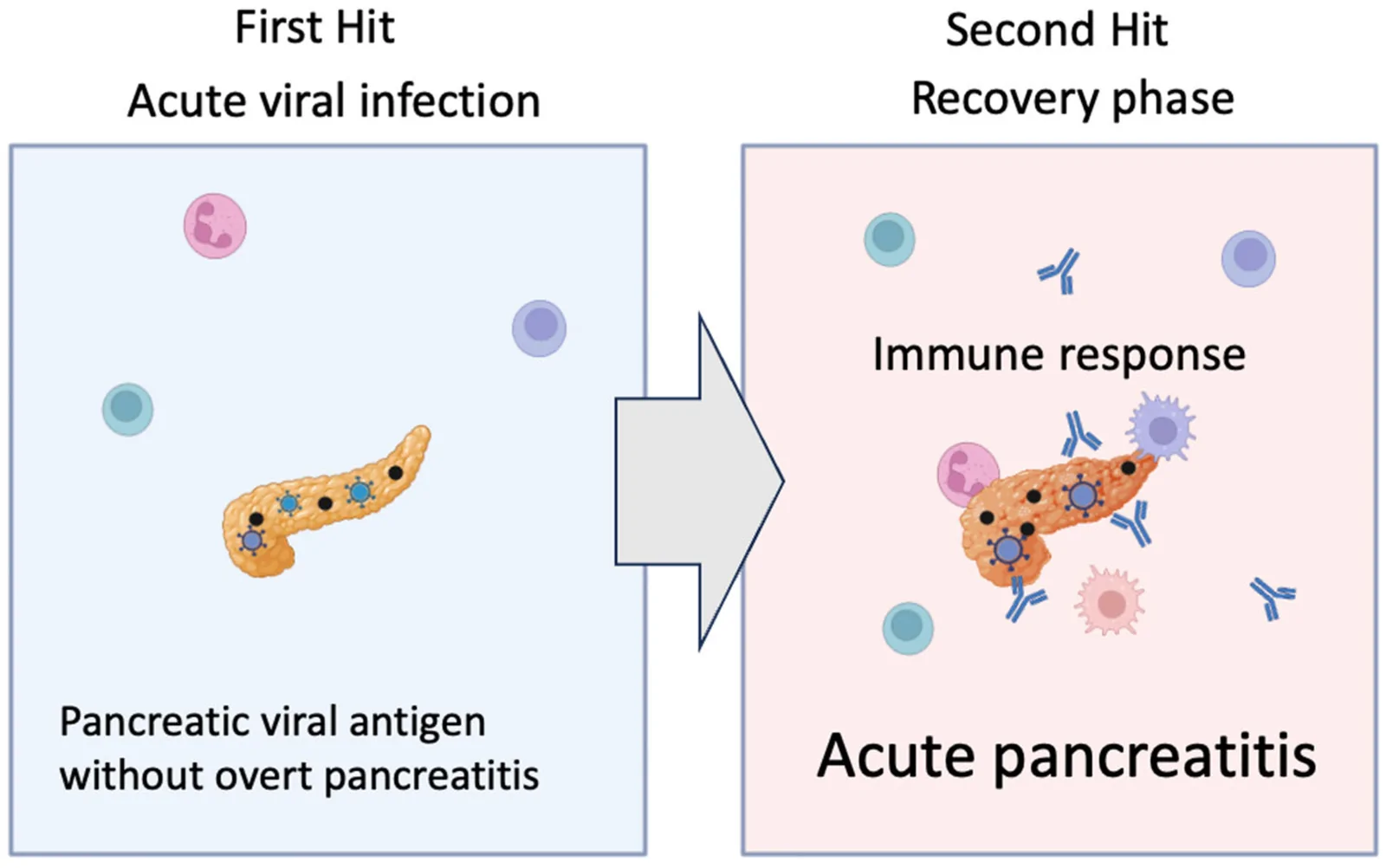
Proposed two-hit model of recovery-phase viral pancreatitis. During the first hit, acute viral infection may result in possible pancreatic viral involvement or antigen persistence without overt pancreatitis. As the systemic manifestations of infection improve, the illness enters the recovery phase. In the proposed second hit, a host immune response may promote pancreatic inflammation in the presence of persistent viral antigens, resulting in acute pancreatitis. This model provides a potential explanation for the occurrence of acute pancreatitis during the recovery phase of viral infection. The proposed mechanism is hypothetical, and neither pancreatic viral involvement nor antigen persistence has been directly demonstrated in most reported cases.

**Table 1:** Reported cases of natural-course recovery-phase viral pancreatitis.

Study	Virus	Age/Sex	Interval from Viral Illness Onset to AP	Recovery Phase at AP Onset
Agarwal et al. [19] (1999)	Hepatitis A virus	7/F	~1 month	Recovering—hepatitis abating; convalescent phase
Amé et al. [20] (2022), Case 2	SARS-CoV-2	65/F	Day 13	Clinically quiescent—asymptomatic preceding infection
Anand et al. [10] (2020)	SARS-CoV-2	59/F	~10 days *	Recovered/improved—discharged †
Cheung et al. [21] (2020)	SARS-CoV-2	38/M	~3 weeks to recurrent AP *	Recovered/improved—clinically stabilized and discharged †
Deniel et al. [22] (2011)	Hepatitis E virus	26/M	~3 weeks	Recovering—hepatitis resolving; liver enzymes near normal
Garty et al. [23] (1995)	Hepatitis A virus	4/M	~2 weeks	Recovering—convalescent phase
Hammami et al. [24] (2019)	Epstein–Barr virus	18/F	NR	Recovering—preceding systemic symptoms; afebrile at AP presentation
Schembri Higgans et al. [25] (2021), Case 2	SARS-CoV-2	87/F	~2 weeks	Clinically quiescent—only mild preceding sore throat; largely asymptomatic
Jeelani et al. [11] (2021)	SARS-CoV-2	24/M	~2 weeks	Recovered—respiratory symptoms resolved
Meireles et al. [26] (2020)	SARS-CoV-2	36/F	Day 11	Recovering—marked clinical improvement; off oxygen
Nguyen & Brophy-Williams [27] (2022)	SARS-CoV-2	9/F	Day 6	Recovered—upper respiratory symptoms resolved
Pinte & Baicus [28] (2020)	SARS-CoV-2	47/M	Day 12	Recovering/recovered—cough faded; laboratory abnormalities normalized
Rajesh et al. [1] (2008), Case 3	Dengue virus	16/F	~3 weeks	Post-acute phase—AP occurred 3 weeks after dengue fever §
Rajesh et al. [1] (2008), Case 5	Mumps virus	47/M	~2 weeks	Post-acute phase—AP occurred 2 weeks after mumps §
Razafindrazoto et al. [29] (2022)	SARS-CoV-2	44/F	~1 week	Recovering—favorable clinical course at home
Samies et al. [30] (2021), Case 3	SARS-CoV-2	16/F	~1 week	Post-symptomatic phase—fever/slight cough occurred ~1 week before AP ‡
Sharma et al. [31] (2021)	SARS-CoV-2	25/F	Day 8	Recovering—fever remitted; other symptoms improved
Suchman et al. [32] (2021)	SARS-CoV-2	16/M	NR	Recovering—severe respiratory illness improved; extubated before AP
Wifi et al. [33] (2021)	SARS-CoV-2	72/F	~1 week	Recovered—initial cough/sneezing completely resolved

† AP developed after hospital discharge and required readmission. ‡ Resolution of the preceding fever and cough was not explicitly stated in the original report. § The original report establishes the interval after the preceding infection but does not explicitly document resolution of the initial symptoms. * Approximate interval based on the clinical sequence reported in the original report. Cases are presented at the patient level. The interval represents the time from onset of the preceding viral illness to AP whenever this could be determined. Recovery status was classified from the clinical course explicitly described in the original reports. Recovered indicates documented resolution of preceding clinical manifestations; Recovering indicates documented clinical improvement or convalescence; Clinically quiescent indicates an asymptomatic or largely asymptomatic preceding infection; and Post-acute/post-symptomatic phase indicates temporal separation from the preceding viral illness when explicit documentation of symptom resolution was unavailable. No recovery status was inferred beyond the information provided in the original reports. Diagnostic evidence, the interval from documented clinical improvement to AP, alternative etiologies, medication exposure, and co-infection status are provided in Supplementary Table S3. AP, acute pancreatitis; NR, not reported.

**Table 2:** Reported cases of recovery-phase pancreatitis following systemic corticosteroid treatment.

Study	Virus	Age/Sex	Interval from Viral Illness Onset to AP	Recovery Phase at AP Onset
AlHarmi et al. [34] (2021)	SARS-CoV-2	52/F	NR *	Recovering—COVID-19 pneumonia resolving; discharge planned
Almutairi et al. [12] (2022)	SARS-CoV-2	71/M	~12 days	Recovered—infection resolved; discharged †
Brikman et al. [35] (2020)	SARS-CoV-2	61/M	~19 days	Recovering—respiratory status improved; off high-flow oxygen
Ghosh et al. [36] (2020)	SARS-CoV-2	63/M	NR	Recovered/improved—discharged; apparently well for 2 days †
Salehi et al. [37] (2022)	SARS-CoV-2	38/M	~2 weeks	Recovered/improved—improved; discharged †
Yamamoto et al. [38] (2021)	SARS-CoV-2	70/F	Day 14	Recovering—COVID-19 symptoms improved
Yu et al. [39] (2026)	SARS-CoV-2	59/F	NR ‡	Recovering—fever resolved; respiratory status improved

† AP developed after hospital discharge and required readmission. * AP was recognized while COVID-19 pneumonia was resolving, but abdominal pain had reportedly begun at least 1 week earlier; therefore, the exact onset of AP could not be determined. ‡ The interval from the onset of COVID-19 illness could not be reliably determined from the original report. Cases are presented at the patient level. These cases developed AP during or after clinical recovery from the preceding viral illness following systemic corticosteroid exposure. The interval represents the time from onset of the preceding viral illness to AP whenever this could be determined from the original report. Recovery status was classified from the clinical course explicitly described in the original reports. Recovered indicates documented resolution of the preceding clinical manifestations, whereas Recovering indicates documented clinical improvement or convalescence. Cases in which the original authors identified a definite alternative etiology of AP were excluded. Detailed corticosteroid regimens, diagnostic evidence, and assessment of alternative etiologies are provided in Supplementary Tables S2 and S3. AP, acute pancreatitis; NR, not reported.

**Table 3:** Conceptual comparison between recovery-phase viral pancreatitis (RVP) and classical immune reconstitution inflammatory syndrome (IRIS). This comparison is conceptual rather than empirical; RVP and classical IRIS have not been directly compared within a single cohort. RVP is proposed to share with classical IRIS a potential temporal relationship between the presence or persistence of pathogen-derived antigens and the subsequent development or recovery of an effective host immune response. However, the two conditions differ in baseline host immune status, the trigger for immune recovery, implicated pathogens, and clinical time course. The proposed mechanism of RVP remains hypothetical, and persistence of pancreatic viral antigens and recovery-associated pancreatic immune activation have not been directly demonstrated in patients with RVP.

Feature	Recovery-Phase Viral Pancreatitis (RVP)	Classical Immune Reconstitution Inflammatory Syndrome (IRIS)
Baseline immune status	Immunocompetent host; no recognized baseline immunodeficiency	Profound, pre-existing immunodeficiency (e.g., HIV infection with markedly depleted CD4+ T-cell count)
Immune trigger	Spontaneous resolution of the acute viral illness; no deliberate immune manipulation	Deliberate therapeutic intervention (antiretroviral therapy initiation or withdrawal of immunosuppression)
Typical pathogens	Common viral pathogens not classically regarded as opportunistic (e.g., SARS-CoV-2, dengue virus, hepatitis A virus, Epstein–Barr virus)	Opportunistic organisms capable of latent or subclinical persistence during immunosuppression (e.g., Mycobacterium tuberculosis, cytomegalovirus, Cryptococcus neoformans, varicella-zoster virus) ^a^
Onset timing	Usually within 1–3 weeks of the onset of the inciting illness; interval from clinical improvement rarely documented (0–7 days when reported)	Weeks to months after antiretroviral therapy initiation
Proposed mechanism	Possible pancreatic viral involvement or antigen persistence (Hit 1), followed by immune recognition during natural recovery (Hit 2)	Restoration of pathogen-specific immunity directed against persistent microbial antigen in a previously immunodeficient host

^a^ Classical IRIS was first described, and remains most extensively characterized, in people living with HIV who initiate antiretroviral therapy; HIV itself is the underlying cause of host immunodeficiency rather than a pathogen targeted by the IRIS reaction. Abbreviations: ART, antiretroviral therapy; CD4+, cluster of differentiation 4-positive; HIV, human immunodeficiency virus; IRIS, immune reconstitution inflammatory syndrome; RVP, recovery-phase viral pancreatitis.

## Data Availability

No new data were created or analyzed in this study. Data supporting the reported findings are available within the cited published articles listed in the References section.

## Supplementary Materials

The supporting information can be downloaded at https://www.mdpi.com/article/10.3390/jcm15207794/s1. Table S1: Detailed characteristics of natural-course recovery-phase viral pancreatitis [1,10,11,19,20,21,22,23,24,25,26,27,28,29,30,31,32,33]; Table S2: Detailed characteristics of recovery-phase pancreatitis following systemic corticosteroid treatment [12,34,35,36,37,38,39]; Table S3: Case-level diagnostic evidence, time intervals, alternative etiologies, medication exposure, and co-infection status in the 26 recovery-phase cases [1,10,11,12,19,20,21,22,23,24,25,26,27,28,29,30,31,32,33,34,35,36,37,38,39]; Table S4: PubMed search strategy.
