# Supplementary material for: Recovery-Phase Viral Pancreatitis (RVP): Proposal of a New Clinical Framework Arising During the Natural Course of Viral Infection
Source: J Clin Med. 2026 Oct 9;15(20):7794. doi: 10.3390/jcm15207794 (PMC13705016; doi:10.3390/jcm15207794)
Supplement: Supplementary file 1 [file jcm-15-07794-s001.zip › jcm-4589736-supplementary.pdf]

Supplementary Materials

Recovery-Phase Viral Pancreatitis: Detailed Case-Level Characteristics

Supplementary Table S1. Detailed characteristics of natural-course recovery-phase viral pancreatitis.

Study	Virus	Age/Sex	Clinical course before AP	Interval from viral illness onset to AP	Setting at AP onset	Evidence supporting AP	Assessment of alternative etiologies	Systemic corticosteroid before AP
Agarwal et al. (1999) [19]	Hepatitis A virus	7/F	Hepatitis abating; authors described convalescent-phase pancreatitis	~1 month	Hospital/NR	Severe abdominal pain/vomiting; amylase 1,560 U/L; US/CT showed enlarged pancreas with focal necrosis	No trauma or medication; lipids/Ca normal; no gallstones or ductal dilatation; weak HEV IgM considered insignificant by authors	Not reported
Amé et al. (2022), Case 2 [20]	SARS-CoV-2	65/F	Asymptomatic infection; remained clinically well before AP	Day 13	At home -> admission	Severe epigastric pain/vomiting; amylase 470 U/L, lipase 1,950 U/L; CT Balthazar C; MRCP normal	No alcohol/toxics/new medication; liver enzymes, lipids, IgG4 and Ca normal; no biliary abnormality on MRCP; microlithiasis not completely excluded because EUS unavailable	Not reported
Anand et al. (2020) [10]	SARS-CoV-2	59/F	COVID-19 improved; discharged	~10 days*	Post-discharge readmission†	Abdominal pain; CT showed marked enlargement of previously atrophic pancreas with diffuse edematous changes suspicious for AP	Prior cholecystectomy; minimal alcohol; authors reported no evident precipitating risk factor	Not reported
Cheung et al. (2020) [21]	SARS-CoV-2	38/M	Initial AP/COVID-19 clinically stabilized; discharged	~3 weeks to recurrent AP*	Post-discharge readmission†	Typical abdominal pain; lipase 10,255 U/L initially and 20,320 U/L at recurrence; CT confirmed recurrent AP	No gallstones/biliary disease; Ca, TG and liver tests normal; no significant alcohol, medications, trauma or surgery; autoimmune work-up unrevealing	Not reported
Deniel et al. (2011) [22]	Hepatitis E virus	26/M	Hepatitis resolving; liver enzymes near normal	~3 weeks	Hospital/NR	Intense epigastric pain; lipase 43× ULN; CT showed grade E necrotizing pancreatitis	No alcohol, drugs, hyperlipidemia, hypercalcemia or trauma; biliary US normal	Not reported
Garty et al. (1995) [23]	Hepatitis A virus	4/M	Convalescent phase of hepatitis	~2 weeks	NR	Severe abdominal pain/vomiting; amylase 4,040 U/L; US showed pancreatic inflammation	No trauma, medications or family history of pancreatic disease; other viral serologies negative; liver/gallbladder US normal	Not reported
Hammami et al. (2019) [24]	Epstein–Barr virus	18/F	Preceding systemic symptoms; afebrile at AP presentation	NR	Admission	Typical abdominal pain; amylase 327 IU/L, lipase 2,016 U/L; CT showed AP	Alcohol, drugs, gallstones, hypercalcemia and hypertriglyceridemia excluded; EBV serology positive	Not reported
Schembri Higgans et al. (2021), Case 2 [25]	SARS-CoV-2	87/F	Mild sore throat; otherwise largely asymptomatic	~2 weeks	Quarantine facility -> hospital	Abdominal pain/vomiting; amylase 499 U/L; CT confirmed AP	Prior cholecystectomy; later MRCP showed no biliary stones; TG only slightly elevated (2.24 mmol/L); long-term low-dose atorvastatin noted as a possible confounder	Not reported
Jeelani et al. (2021) [11]	SARS-CoV-2	24/M	Respiratory symptoms resolved spontaneously	~2 weeks	Admission	Epigastric pain; lipase 2,025 U/L, later 4,174 U/L; CT showed moderately severe AP	Alcohol/drug screen negative; TG and Ca normal; no gallstones; IgG4 normal; no current medications	Not reported
Meireles et al. (2020) [26]	SARS-CoV-2	36/F	Marked clinical/laboratory/radiological improvement; oxygen discontinued	Day 11	During hospitalization	Belt-like epigastric pain; amylase/lipase >10× ULN; clinically diagnosed AP	Gallstones, alcohol/drugs, trauma, hyperTG, hyperCa, autoimmunity and other infections excluded	No steroid therapy
Nguyen & Brophy-Williams (2022) [27]	SARS-CoV-2	9/F	Upper respiratory symptoms resolved	Day 6	At home -> admission	Sudden severe epigastric pain/vomiting; lipase 13,500 U/L; CT consistent with AP	No alcohol/drugs/medications/trauma/family history; no gallstones; Ca/TG normal; other acute viral serologies unrevealing	Not reported
Pinte & Baicus (2020) [28]	SARS-CoV-2	47/M	Cough faded; laboratory abnormalities normalized	Day 12	Outpatient course -> admission	Severe epigastric pain; lipase 22× ULN, amylase 6× ULN; CT showed pancreatic edema	Gallstones/biliary obstruction, alcohol, smoking, drugs, trauma, hyperTG and hyperCa excluded	Not reported
Rajesh et al. (2008), Case 3 [1]	Dengue virus	16/F	Post-acute phase after dengue fever	~3 weeks	NR	Pancreatic-type pain; amylase 439 U/L; CT showed enlarged pancreas with peripancreatic collection	No alcohol or smoking; family history of chronic pancreatitis noted	Not reported
Rajesh et al. (2008), Case 5 [1]	Mumps virus	47/M	Post-acute phase after mumps infection	~2 weeks	NR	Amylase 781 U/L; contrast CT showed Balthazar grade C AP	No other apparent cause; beer use without binge drinking; no smoking or family history of pancreatitis	Not reported
Razafindrazoto et al. (2022) [29]	SARS-CoV-2	44/F	Favorable clinical course at home	~1 week	At home -> admission	Severe epigastric pain; lipase 301 U/L (>3× ULN); CT showed edematous pancreatitis	Lithiasis, alcohol, hyperCa, hyperTG, tumor, trauma and surgery excluded	No corticosteroid reported
Samies et al. (2021), Case 3 [30]	SARS-CoV-2	16/F	Subjective fever/slight cough ~1 week earlier; no respiratory distress at AP presentation	~1 week	Admission	Epigastric pain radiating to back; lipase 1,909 U/L; US showed prominence of pancreatic head/tail/duct	Previous AP >1 year earlier; genetic evaluation negative; single gallstone present; alternative etiology therefore not completely excluded	Not reported
Sharma et al. (2021)	SARS-CoV-2	25/F	Fever remitted; other symptoms improved	Day 8	Home isolation -> admission	Severe epigastric pain radiating to back/vomiting; lipase 11,920.7 U/L, amylase	No alcohol, recent abdominal surgery, trauma or known pancreatitis-inducing drugs; TG 97.4 mg/dL	Not reported

Study	Virus	Age/Sex	Clinical course before AP	Interval from viral illness onset to AP	Setting at AP onset	Evidence supporting AP	Assessment of alternative etiologies	Systemic corticosteroid before AP
[31]						1,814.6 U/L; CECT showed acute interstitial pancreatitis with mild ascites (CTSI 6)		
Suchman et al. (2021) [32]	SARS-CoV-2	16/M	Severe respiratory illness improved; extubated before AP diagnosis	NR	During hospitalization	Epigastric pain with elevated lipase; classified as idiopathic AP in the original study	No etiology identified in chart review	Not reported
Wifi et al. (2021) [33]	SARS-CoV-2	72/F	Initial respiratory symptoms completely resolved	~1 week	At home -> admission	Abdominal pain, nausea/vomiting; amylase 1,667 U/L, lipase 710 U/L; diagnosed AP	Other causes reported excluded; TG 81 mg/dL, Ca 8.6 mg/dL	Not reported

* Approximate interval based on the clinical sequence reported in the original article. † AP developed after hospital discharge and required readmission.

Legend: Cases are presented at the patient level. Natural-course RVP denotes recovery-phase AP without documented preceding systemic corticosteroid exposure. Timing, recovery status, diagnostic evidence, and alternative-etiology assessment were based on information reported in the original articles and were not strengthened by inference. “Not reported” indicates that systemic corticosteroid use before AP was not documented in the report. Reference numbers correspond to the main manuscript. AP, acute pancreatitis; Ca, calcium; CECT, contrast-enhanced computed tomography; CT, computed tomography; CTSI, CT severity index; EUS, endoscopic ultrasonography; HEV, hepatitis E virus; MRCP, magnetic resonance cholangiopancreatography; NR, not reported; TG, triglycerides; ULN, upper limit of normal; US, ultrasonography.

Supplementary Table S2. Detailed characteristics of recovery-phase pancreatitis following systemic corticosteroid treatment.

Study	Virus	Age/Sex	Clinical course before AP	Interval from viral illness onset to AP	Setting at AP onset	Evidence supporting AP	Systemic corticosteroid exposure	Relationship of corticosteroid exposure to AP	Assessment of alternative etiologies
AlHarmi et al. (2021) [34]	SARS-CoV-2	52/F	COVID-19 pneumonia was resolving, and discharge was being planned; exit nasopharyngeal swabs were negative on days 17 and 18.	NR*	During hospitalization	CT: early acute pancreatitis with peripancreatic fat stranding/fluid; no necrosis. Serum lipase unavailable; amylase 47 U/L.	Dexamethasone 6 mg IV daily ×2 d → methylprednisolone 40–60 mg IV q8h ×8 d → dexamethasone 6 mg PO daily ×5 d (total steroid course 15 d).	Steroid exposure overlapped the period before AP. Authors explicitly noted that several administered drugs, including dexamethasone, could have caused drug-induced AP.	Alcohol denied; prior cholecystectomy; repeat TG near normal; no recent ERCP/surgery, trauma, anatomic anomaly, or pancreatic cancer.
Almutairi et al. (2022) [12]	SARS-CoV-2	71/M	Hospitalized for COVID-19 pneumonia for 10 days; discharged with resolution of the infection.	~12 days	Post-discharge readmission†	Epigastric pain; lipase 1,023 U/L, amylase 544 U/L; contrast CT showed inflammatory changes around pancreatic head and tail.	Dexamethasone was administered during the 10-day COVID-19 admission; dose/duration not reported.	AP occurred shortly after a hospitalization that included dexamethasone; the report attributed AP to previous COVID-19 infection rather than establishing steroid causality.	No alcohol history; TG 1.78 mmol/L, Ca 2.47 mmol/L; US/CT showed no gallstones, cholecystitis, or biliary dilatation.
Brikman et al. (2020) [35]	SARS-CoV-2	61/M	Severe COVID-19 pneumonia; respiratory status improved after 7 days and high-flow oxygen was no longer required.	~19 days	During hospitalization	Sudden abdominal pain; lipase 203 U/L (<3× ULN), amylase 142 U/L; contrast CT showed pancreatitis; met 2/3 Atlanta criteria.	Dexamethasone 10 mg IV twice daily ×5 d with tapering thereafter; also received tocilizumab and multiple other drugs.	Authors considered SARS-CoV-2 infection, medications, or a combination of both. Dexamethasone and other administered drugs were recognized as pancreatitis-associated.	No gallstones/biliary abnormality; no heavy alcohol use; Ca/liver enzymes normal; TG was normal 1 day before AP and only mildly elevated around AP.
Ghosh et al. (2020) [36]	SARS-CoV-2	63/M	Hospitalized with COVID-19 and treated with remdesivir, anticoagulant, and corticosteroids; discharged, then apparently well for 2 days.	NR	Post-discharge readmission†	CT: bulky pancreatic tail with <30% necrosis; lipase 412 U/L; managed as pancreatitis.	Corticosteroids were given during the preceding COVID-19 hospitalization; agent, dose, and duration not reported.	Steroid exposure preceded AP; the report did not establish a steroid contribution and considered COVID-19-related pancreatitis.	No history of alcohol, hypertriglyceridemia, or cholelithiasis; authors stated absence of a known etiology.
Salehi et al. (2022) [37]	SARS-CoV-2	38/M	Hospitalized for COVID-19 pneumonia, improved, and was discharged without complications or medications.	~2 weeks	Post-discharge readmission†	Severe epigastric pain radiating to back; amylase 773 U/L, lipase 286 U/L; US/CT showed severe necrotizing pancreatitis (>30% necrosis).	Dexamethasone 8 mg every 12 h during the preceding COVID-19 hospitalization; exact treatment duration not stated.	Steroid exposure preceded AP, but the authors reported exclusion of drugs as a potential cause and attributed the episode to recent COVID-19.	Alcohol, drugs, hyperTG, hyperCa, hepatitis B/HIV, prior/familial pancreatitis and trauma were reported as excluded.
Yamamoto et al. (2021) [38]	SARS-CoV-2	70/F	COVID-19 symptoms improved after treatment with remdesivir and dexamethasone.	Day 14	During hospitalization	Abdominal tenderness with elevated pancreatic enzymes; CT showed enlarged pancreatic parenchyma; diagnosed AP.	Dexamethasone 6 mg plus remdesivir; exact steroid duration not stated.	Authors explicitly stated that dexamethasone and remdesivir might have been involved because AP occurred after their administration.	History of cholecystectomy; other etiologic evaluation was not detailed in this short report.
Yu et al. (2026) [39]	SARS-CoV-2	59/F	COVID-19 pneumonia treated after extubation; fever resolved and respiratory status improved by day 18.	NR‡	During hospitalization	Amylase 503 U/L and lipase 353 U/L (>3× ULN); CT showed peripancreatic inflammation; mild interstitial edematous AP.	Dexamethasone 10 mg IV daily ×3 d, then 5 mg daily ×2 d, starting day 13; also received nirmatrelvir/ritonavir.	AP appeared at/just after completion of the 5-day dexamethasone course. Authors considered drug-related mechanisms but concluded no single cause could be identified, and AP was likely multifactorial.	TG and Ca normal; alcohol denied; CT showed no gallstones/sludge/bile duct dilation, although occult biliary disease could not be completely excluded; altered post-Whipple anatomy and multiple comorbidities.

* AP was recognized while COVID-19 pneumonia was resolving, but abdominal pain had reportedly begun at least 1 week earlier; therefore, the exact AP onset could not be determined. † AP developed after hospital discharge and required readmission. ‡ The interval from onset of COVID-19 illness could not be reliably determined from the original report.

Legend: These cases developed AP during or after clinical recovery from the preceding viral illness after systemic corticosteroid exposure and were analyzed separately from natural-course RVP. Inclusion does not establish corticosteroid or viral causality. Cases in which the original authors identified a definite alternative etiology of AP were excluded from this table. Reference numbers correspond to the main manuscript. AP, acute pancreatitis; Ca, calcium; CT, computed tomography; ERCP, endoscopic retrograde cholangiopancreatography; IV, intravenous; NR, not reported; PO, oral; TG, triglycerides; ULN, upper limit of normal; US, ultrasonography.

Supplementary Materials

Supplementary Table S3. Case-level diagnostic evidence, time intervals, alternative etiologies, medication exposure, and co-infection status in the 26 recovery-phase cases.

Case [ref]	Virus	AP diagnosis	Diagnostic evidence reported	Viral illness onset to AP	Clinical improvement or discharge to AP	Alternative causes evaluated (as reported)	Medication exposure before AP	Co-infection	Original authors' assessment
**Natural-course recovery-phase viral pancreatitis (n = 19; Table 1)**									

Case [ref]	Virus	AP diagnosis	Diagnostic evidence reported	Viral illness onset to AP	Clinical improvement or discharge to AP	Alternative causes evaluated (as reported)	Medication exposure before AP	Co-infection	Original authors' assessment
Agarwal et al., 1999 [19]	Hepatitis A virus	Fulfilled	Acute abdominal pain with marked tenderness; amylase 1560 U/L (35–127); US and CT: enlarged pancreas with focal necrosis	~1 month	NR	No trauma, medication, worm infestation, or haemolysis; lipid profile and calcium normal; no gallstones or ductal dilatation (US)	None reported	Blood and urine cultures and Widal test negative; anti-HEV IgM weakly reactive (considered not significant by the authors)	Pancreatitis during the convalescent phase of cholestatic hepatitis A
Amé et al., 2022, Case 2 [20]	SARS-CoV-2	Fulfilled	Severe epigastric pain; lipase 1950 U/L, amylase 470 U/L (ULN NR); CT: AP (Balthazar C)	Day 13	NA (asymptomatic infection)	No gallstones or ductal abnormality (MRCP); triglycerides, calcium, and IgG4 normal; no alcohol or toxics; active smoker	Usual medication only (not specified); no new medication	NR	AP as the main manifestation of SARS-CoV-2 infection; possible role of active smoking raised
Anand et al., 2020 [10]	SARS-CoV-2	Fulfilled	Abdominal pain; amylase not measured; CT: markedly enlarged pancreas with diffuse oedematous changes	~10 days	5 days after discharge	Previous cholecystectomy; minimal alcohol use; no precipitating risk factors	Intravenous vancomycin; doxycycline after discharge	Streptococcal pneumonia	Idiopathic AP; possible association with COVID-19
Cheung et al., 2020 [21]	SARS-CoV-2	Fulfilled	Recurrent epigastric pain; lipase 20,320 (units as reported; ULN NR); CT and MRI: AP	~3 weeks (recurrent AP)	~1 week after discharge from the initial AP episode	Calcium, triglycerides, bilirubin, and liver tests normal; no gallstones (US, MRCP); no alcohol, medication, trauma, or surgery; hepatitis serology and IgG4 unremarkable	None	NR	Presumptive COVID-19-associated recurrent AP
Deniel et al., 2011 [22]	Hepatitis E virus	Fulfilled	Intense epigastric pain; lipase 43 × ULN; CECT: necrotizing pancreatitis	~3 weeks	NR	No alcohol, drugs, hyperlipidaemia, hypercalcaemia, or trauma; biliary US normal	None reported	HAV, HBV, HCV, CMV, and EBV negative	Complication of acute hepatitis E
Garty et al., 1995 [23]	Hepatitis A virus	Fulfilled	Severe abdominal pain; amylase 4040 U/L (50–250); US: increased pancreatic echogenicity and peritoneal fluid	~2 weeks	NR	No trauma, medication, or family history of pancreatic disease; normal gallbladder (US)	None	HBV, HCV, mumps, measles, EBV, CMV, coxsackie, and varicella serology negative	Pancreatitis in the convalescent period of hepatitis A infection
Hammami et al., 2019 [24]	Epstein–Barr virus	Fulfilled	Upper abdominal pain; lipase 2016 U/L, amylase 327 IU/L (ULN NR; all three criteria fulfilled according to the authors); CT: AP	NR	NR	Alcohol, gallstones, drugs, hypercalcaemia, and hypertriglyceridaemia excluded	None	HAV, HBV, HCV, HIV, and CMV negative	EBV-associated AP
Schembri Higgans et al., 2021, Case 2 [25]	SARS-CoV-2	Fulfilled	Generalized abdominal pain with epigastric/left upper quadrant tenderness; amylase 499 U/L (ULN NR; lipase unavailable); CT: AP	~2 weeks	NR	Previous cholecystectomy; no biliary stones on later MRCP; triglycerides 2.24 mmol/L (ULN 2.2)	Long-term low-dose atorvastatin; co-amoxiclav and clarithromycin, then piperacillin-tazobactam	Probable (chest crepitations and rising inflammatory markers; antibiotics escalated)	Linked to COVID-19; mildly raised triglycerides and statin use noted as possible

Case [ref]	Virus	AP diagnosis	Diagnostic evidence reported	Viral illness onset to AP	Clinical improvement or discharge to AP	Alternative causes evaluated (as reported)	Medication exposure before AP	Co-infection	Original authors' assessment
									confounders
Jeelani et al., 2021 [11]	SARS-CoV-2	Fulfilled	Epigastric pain; lipase 2025 U/L (73–393); CECT: moderately severe AP	~2 weeks	NR	Triglycerides, calcium, blood alcohol, and IgG4 normal; no gallstones or ductal dilatation (US); no current medications	None (empirical piperacillin-tazobactam after AP onset)	Blood cultures and respiratory panel negative except SARS-CoV-2	AP associated with COVID-19 after resolution of respiratory symptoms
Meireles et al., 2020 [26]	SARS-CoV-2	Fulfilled	Belt-like epigastric pain; amylase 718 U/L and lipase 631 U/L (~10-fold elevation); CT: no pancreatic abnormalities	Day 11	NR	Alcohol, drugs, trauma, hypertriglyceridaemia, hypercalcaemia, autoimmunity, and other infections excluded; no cholelithiasis (US); stage V chronic kidney disease	Nifedipine, carvedilol; no corticosteroids	Coxsackie, HSV, and CMV IgM negative; procalcitonin 5.61 at admission (as reported)	Acalculous AP in COVID-19; immune-mediated mechanism suggested
Nguyen & Brophy-Williams, 2022 [27]	SARS-CoV-2	Fulfilled	Severe epigastric pain; lipase 13,500 U/L (ULN NR; all three criteria fulfilled according to the authors); CT: bulky heterogeneous pancreas with ascites	Day 6	NR	No alcohol, drugs, medication, trauma, or personal/family history; no gallstones (US); calcium and triglycerides normal	None	Serology did not suggest acute mumps, VZV, CMV, EBV, or HSV infection	SARS-CoV-2 as a potential cause of AP
Pinte & Baicus, 2020 [28]	SARS-CoV-2	Fulfilled	Severe epigastric pain radiating to the back; lipase 22 × ULN, amylase 6 × ULN; CT: oedematous changes in the pancreatic tail	Day 12	NR	Gallstones/biliary obstruction, alcohol, smoking, drugs, trauma, hypertriglyceridaemia, and hypercalcaemia excluded	No home medication	Procalcitonin normal; patient-level infectious work-up NR	SARS-CoV-2 infection might be responsible
Rajesh et al., 2008, Case 3 [1]	Dengue virus	Fulfilled	Pancreatic-type pain; amylase 439 U/L (28–100); CECT: enlarged pancreas with peripancreatic collection	~3 weeks	NR	No alcohol or smoking; occasional cassava intake; father with chronic pancreatitis and diabetes	NR	NR	AP complicating viral infection without another apparent cause
Rajesh et al., 2008, Case 5 [1]	Mumps virus	Fulfilled	Admitted with AP; amylase 781 U/L (28–100); CECT: AP (Balthazar C)	~2 weeks	NR	No other cause apparent; beer intake without binge drinking; significant cassava intake; orchitis developed subsequently; later recurrent AP with pancreatic calculi	NR	NR	AP complicating mumps; the authors noted that first attacks might alternatively represent idiopathic pancreatitis occurring during viral infection
Razafindrazoto et al., 2022 [29]	SARS-CoV-2	Fulfilled	Severe epigastric pain; lipase 301 U/L (0–60); CT: oedematous pancreatitis without gallstones	~1 week	NR	Lithiasis (US, CT), alcohol, hypercalcaemia, hypertriglyceridaemia, tumour, trauma, and surgery excluded;	Paracetamol, aspirin, atorvastatin, amoxicillin-clavulanic acid, prophylactic	None documented (empirical antibiotics); HBV, HCV, HAV IgM,	COVID-19-induced AP

Case [ref]	Virus	AP diagnosis	Diagnostic evidence reported	Viral illness onset to AP	Clinical improvement or discharge to AP	Alternative causes evaluated (as reported)	Medication exposure before AP	Co-infection	Original authors' assessment
						triglycerides 2.1 g/L (0–1.99)	enoxaparin	and HIV negative	
Samies et al., 2021, Case 3 [30]	SARS-CoV-2	Fulfilled	Epigastric pain radiating to the back; lipase 1909 U/L (4–39); US: prominence of the pancreatic head, tail, and duct	~1 week	NR	Single gallstone (US); pancreatitis >1 year earlier; genetic evaluation negative; triglycerides normal; complete medication history not obtained	NR	NR	Temporal association with SARS-CoV-2; other etiologies difficult to rule out
Sharma et al., 2021 [31]	SARS-CoV-2	Fulfilled	Epigastric pain radiating to the back; lipase 11,920.7 U/L (23–300), amylase 1814.6 U/L (30–110); CECT: acute interstitial pancreatitis	Day 8	NR	No alcohol, recent surgery, trauma, or known offending drugs; no gallstones (US); triglycerides 97.4 mg/dL	Doxycycline, favipiravir, ivermectin, montelukast-levocetirizine, multivitamins, zinc	None documented (empirical antibiotics)	COVID-19-induced AP
Suchman et al., 2021, Patient 13 [32]	SARS-CoV-2	Fulfilled	Abdominal pain; lipase 233.3 U/L (12–70)	NR	NR (extubated before AP diagnosis)	No etiology identified (idiopathic)	NR	NR (registry-based report)	Idiopathic AP in a child hospitalized with COVID-19
Wifi et al., 2021 [33]	SARS-CoV-2	Fulfilled	Severe diffuse abdominal pain; amylase 1667 U/L (0–140), lipase 710 U/L (0–60); abdominal CT normal	~1 week	NR	Calcium and triglycerides normal; other causes excluded according to the authors	Beta-blockers, aspirin, nitrates; paracetamol and nasal decongestants	NR	COVID-induced pancreatitis
**Steroid-associated recovery-phase pancreatitis (n = 7; Table 2)**									
AlHarmi et al., 2021 [34]	SARS-CoV-2	Fulfilled	Epigastric pain (present for ≥1 week); amylase 47 U/L (30–118), lipase not available; CT: early AP	NR	NR	Triglycerides 3.4 then 1.9 mmol/L; no ERCP or recent surgery; cholecystectomy 10 years earlier; no anatomical anomaly	Dexamethasone, methylprednisolone, ceftriaxone, doxycycline, azithromycin, enoxaparin, proton pump inhibitors, metoclopramide, and others	None documented (empirical antibiotics)	Association with COVID-19 possible; drug-induced AP possible
Almutairi et al., 2022 [12]	SARS-CoV-2	Fulfilled	Abdominal pain with epigastric tenderness; lipase 1023 U/L, amylase 544 U/L (ULN NR); CT: AP	~12 days	2 days after discharge	No gallstones (US, CT); calcium normal; triglycerides 1.78 mmol/L; no alcohol; end-stage renal disease on haemodialysis	Piperacillin-tazobactam, dexamethasone; regular insulin, valsartan, levothyroxine, alfacalcidol, calcium carbonate	None documented (empirical antibiotics)	COVID-19-induced AP after resolution of infection
Brikman et al., 2020 [35]	SARS-CoV-2	Fulfilled	Sudden diffuse abdominal pain; lipase 203 U/L (21–67), amylase 142 U/L (28–100); CECT: AP	~19 days	NR	Liver tests and calcium normal; no gallstones; no heavy alcohol use; triglycerides 3.18 mmol/L (normal 1 day earlier)	Azithromycin, hydroxychloroquine, zinc, tocilizumab, dexamethasone, enoxaparin, lopinavir/ritonavir, pantoprazole, ciprofloxacin, clindamycin	*Staphylococcus aureus* (sputum culture)	SARS-CoV-2 infection, medications, or both
Ghosh et al., 2020	SARS-CoV-	As reported by the original	Lipase 412 U/L (73–160), amylase 58 U/L (25–115); non-contrast CT: bulky	NR	NR	No alcohol, hypertriglyceridaemia, or	Metformin; intravenous antibiotics, remdesivir,	*Klebsiella pneumoniae* urinary tract infection	COVID-19-induced AP with

Case [ref]	Virus	AP diagnosis	Diagnostic evidence reported	Viral illness onset to AP	Clinical improvement or discharge to AP	Alternative causes evaluated (as reported)	Medication exposure before AP	Co-infection	Original authors' assessment
[36]	2	authors	pancreatic tail with focal necrosis (<30%)			cholelithiasis	anticoagulant, corticosteroids, insulin	(same admission)	pancreatic necrosis
Salehi et al., 2022 [37]	SARS-CoV-2	Fulfilled	Severe epigastric pain radiating to the back; amylase 773 U/L (30–110), lipase 286 U/L (0–160); US and CT: necrotizing pancreatitis	~2 weeks	1 week after discharge	Alcohol, drugs, hypertriglyceridaemia, hypercalcaemia, HBV, HIV, previous attacks, family history, and trauma excluded	Remdesivir, dexamethasone	NR	COVID-19-induced AP
Yamamoto et al., 2021 [38]	SARS-CoV-2	As reported by the original authors	Abdominal tenderness; amylase 238 U/L, lipase 381 U/L (ULN NR); CT: enlarged pancreatic parenchyma	Day 14	NR	History of cholecystectomy; obesity, diabetes, dyslipidaemia	Remdesivir, dexamethasone 6 mg	NR	AP related to COVID-19; dexamethasone and remdesivir might have been involved
Yu et al., 2026 [39]	SARS-CoV-2	Fulfilled	Epigastric tenderness; amylase 503 U/L (35–135), lipase 353 U/L (8–53); CT: peripancreatic inflammation	NR	0 days (onset on the day of fever resolution and respiratory improvement)	Triglycerides and calcium normal; no alcohol; no cholelithiasis, sludge, or ductal dilatation (CT; MRCP/EUS not feasible); previous pancreaticoduodenectomy	Voriconazole, piperacillin-tazobactam, nirmatrelvir/ritonavir, dexamethasone	*Escherichia coli* and *Enterococcus faecium* (sputum mNGS); chronic pulmonary aspergillosis	AP temporally associated with COVID-19 with multiple competing risk factors

AP diagnosis: "Fulfilled" indicates that at least two of the three revised Atlanta criteria (characteristic abdominal pain; serum amylase or lipase ≥3 times the upper limit of normal; characteristic imaging findings) were documented in the original report. "As reported by the original authors" indicates that the diagnosis of acute pancreatitis was accepted as reported in the original publication because not all components of the revised Atlanta criteria were documented or assessable. Values are reproduced as reported; reference ranges are shown when given in the original report.

Intervals were recorded as stated in, or directly derivable from the clinical sequence reported in, the original reports; when the timing was not reported or was ambiguous, the interval is shown as NR and was not estimated. The interval from clinical improvement or discharge to AP was explicitly reported in 5 of 26 cases.

All information in this table is reproduced from the original reports without additional interpretation.

Abbreviations: AP, acute pancreatitis; CECT, contrast-enhanced computed tomography; CT, computed tomography; MRCP, magnetic resonance cholangiopancreatography; mNGS, metagenomic next-generation sequencing; NA, not applicable; NR, not reported; TG, triglycerides; ULN, upper limit of normal; US, ultrasonography.

Supplementary Table S4. PubMed search strategy.

Database: PubMed/MEDLINE

Search terms entered: Acute pancreatitis viral infection case report

Field tags and filters: None (PubMed automatic term mapping)

Date of last search: July 20, 2026; records retrieved: 671

Reproduction: Restricting the Entrez date to July 20, 2026 or earlier reproduces the same 671 records.

Full query as translated by PubMed:

```
("pancreatitis"[MeSH Terms] OR "pancreatitis"[All Fields] OR ("acute"[All Fields] AND "pancreatitis"[All Fields]) OR "acute pancreatitis"[All Fields])

AND ("virus diseases"[MeSH Terms] OR ("virus"[All Fields] AND "diseases"[All Fields]) OR "virus diseases"[All Fields] OR ("viral"[All Fields] AND "infection"[All Fields]) OR "viral infection"[All Fields])

AND ("case reports"[Publication Type] OR "case report"[All Fields])

AND 1800/01/01:2026/07/20[Date - Entry]
```

Because PubMed automatically explodes MeSH terms, the search also captured reports indexed under specific viral diseases (e.g., COVID-19, hepatitis A, mumps, and dengue) subsumed under “Virus Diseases”.

Citation searching: backward citation searching (reference lists) and forward citation searching (Google Scholar “Cited by” and Web of Science “Times Cited”) of the 26 recovery-phase reports identified no additional eligible cases. Google Scholar and Web of Science were otherwise used only for full-text retrieval.
